# Characterization of a Complex Mixture of Immunomodulator Peptides Obtained from Autologous Urine

**DOI:** 10.1155/2020/3683782

**Published:** 2020-04-02

**Authors:** Alberto Fragoso, Mérida Pedraza-Jiménez, Laura Espinoza-González, María Luisa Ceja-Mendoza, Hugo Sánchez-Mercado, Gloria Robles-Pérez, Julio Granados, Emilio Medina-Rivero

**Affiliations:** ^1^Instituto de Alergias y Autoinmunidad Dr. Maximiliano Ruiz Castañeda A.C., Luisa Isabel Campos #16, col. Revolución, Acambay, Edo. de México 50300, Mexico; ^2^Departamento de Trasplantes, Instituto Nacional de Ciencias Médicas y Nutrición Salvador Zubirán, Ciudad de México, Mexico; ^3^Unidad de Desarrollo e Investigación en Bioprocesos (UDIBI), Escuela Nacional de Ciencias Biológicas, Instituto Politécnico Nacional, Ciudad de México 11340, Mexico

## Abstract

A complex mixture of peptides plays a key role in the regulation of the immune system; different sources as raw materials mainly from animals and vegetables have been reported to provide these extracts. The batch-to-batch product consistency depends on in-process controls established. However, when an immunomodulator is a customized product obtained from the same volunteer who will receive the product to personalize the treatment, the criteria to establish the consistency between volunteers are different. In this sense, it is expected to have the same molecular weight range although the profile of peptide abundance is different. Here, we characterized the peptide profile of three extracts of an immunomodulator obtained from the urine of different volunteers suffering from three different diseases (i.e., allergic rhinitis, rheumatoid arthritis, and chronic rhinopharyngitis), using size exclusion chromatography (SEC) and mass spectrometry (MS). The peptides contained in the immunomodulators were stable after six months, stored in a refrigerator. Our results showed a chromatographic profile with the same range of low molecular weight (less than 17 kDa) in all analyzed samples by SEC; these results were also confirmed by MS showing an exact mass spectrum from 3 to 13 kDa. The fact that the peptide profiles were conserved during a six-month period at refrigeration conditions (2 to 8°C) maintaining the quality and stability of the immunomodulator supports the notion that it might be an alternative in the treatment of chronic hypersensibility disorders.

## 1. Introduction

Biological extracts containing a complex mixture of peptides represent an alternative for the treatment of chronic-degenerative diseases. This peptide distribution promotes the modulation of the immune response by a different mechanism of action still not fully understood, yet they have shown therapeutic properties and therefore represent an improvement in certain diseases [1, 2]. For this reason, it is essential to demonstrate consistency in the physicochemical properties of the product in order to obtain the expected response. Previous studies on similar products containing peptide distribution such as transfer factor (Transferon®) or collagen hydrolysate (Colagenart®) showed a high reproducibility in the physicochemical properties among batches. Transferon® is used as a drug product for the treatment of chronic-degenerative diseases, while Colagenart® is prescribed as a dietary supplement, which have been used as a viable alternative to preserve joint and skin health [3–5].

Nowadays, personalized medicine, also known as individualized medicine or precision medicine, is becoming an innovative treatment in critical areas such as oncology. This approach, based on vaccination, consists on reinjecting a sample from the patient's own immunomodulator, after sample processing, to obtain a fraction of them that contains the peptide distribution which will be presented as own antigens to boost the immune response and serve as an immunomodulator and, therefore, might also be useful in people suffering from autoimmune diseases or in certain innate or adaptive immunodeficiencies [6–12].

This complex mixture of peptides, for reinjecting, is extracted mainly from blood cells or tissues (e.g., solid tumors) [13] and then extracted by standard separation bioprocesses such as precipitation, filtration, or chromatography, followed by formulation, sterile filtration, and the filling of the vials.

Peptides obtained from urine have been used for the treatment of patients with various forms of hypersensitivity (allergies of the skin and mucous membranes) like asthma and various forms of arthritis, for the last 40 years, showing a remarkable clinical improvement within the first weeks of treatment that last for months or even years particularly in patients with early diagnosis [7, 8]. Since the scientist Ruiz Castañeda developed the process to obtain peptides from urine, it allowed to invent the immunomodulator administered by injection or via oral for the treatment of various pathologies, such as autoimmune and allergic diseases. It was possible due to the experience accumulated in medicine, immunology, and bacteriology, which was obtained during his stay in the National School of Medicine, University of Paris, Pasteur Institute, and Harvard [14].

Since 1940, endogenous intradermal injection of the peptides extracted from autologous urine has been used for the treatment of chronic immunologic disorders; according to a repeated dose scheme during 6 months, the results have been proven to be safe (no adverse effects have been reported) and effective mainly in the treatment of allergies of the skin and mucosa, reactive arthritis, and psoriasis [14].

In this study, the distribution of a complex mixture of peptides extracted from human urine in 3 patients was characterized, one with allergic rhinitis, a second one with arthritis rheumatoid, and a third one with chronic rhinopharyngitis. All three patients used the formulation in weekly doses (once a week for six months). For peptide analysis, cutting-edge technology was employed and included size exclusion chromatography and mass spectrometry. Additionally, peptide stability (at 5 ± 3°C) during a 6-month period was demonstrated.

## 2. Materials and Methods

### 2.1. Samples and Reagents

Three samples of immunomodulator obtained from patients with allergic rhinitis, rheumatoid arthritis, and chronic rhinopharyngitis were provided by Instituto de Alergias y Autoinmunidad Dr. Maximiliano Ruiz Castañeda A.C. (Mexico City, Mexico). The immunomodulators were obtained by a bioprocess containing several steps that include selective precipitation of low molecular peptides with organic solvents [14]. Sodium chloride, and monobasic, dibasic sodium phosphate, and sodium 3-(trimethylsilyl)tetradeuteriopropionate (TSP) were obtained from J. T. Baker and (NY, USA) Sigma-Aldrich (MO, USA). Mass spectrometry grade water, formic acid, and acetonitrile were obtained from Sigma-Aldrich (MO, USA).

### 2.2. Methods

#### 2.2.1. Size Exclusion Chromatography (SEC)

A size exclusion chromatography analysis (SE-UPLC) was performed according to Medina-Rivero et al. [3]. Briefly, 10 *μ*L of each immunomodulator was injected in a Waters® BEH 125 SEC column (1.7 *μ*m × 4.6 × 150 mm) using an Acquity UPLC Class-H system (Waters; MA, USA) with UV detection. A 50 mM phosphate-buffered solution (pH 6.8) was used as mobile phase. Data was acquired and processed with the Empower® software (Waters®).

#### 2.2.2. Mass Spectrometry (MS)

MS analyses were performed according to our previous studies for the analysis of the quality attributes of complex molecules in order to demonstrate batch-to-batch consistency [4, 5]. In brief, peptide samples were separated and in-line desalted using a Waters® CSH C18 reverse phase column (1.7 *μ*m, 2.1 × 150 mm) and a gradient from 0% to 25% of Acetonitrile with formic acid (0.1%) as a mobile phase. Analyte ions were obtained by electrospray ionization and analyzed using a quadrupole–time-of-flight (MS-Q-Tof) Vion® spectrometer coupled to an Acquity UPLC class H chromatograph (Waters®). Data was acquired and processed with the UNIFI® software (Waters).

#### 2.2.3. Stability Test

A long-term stability test was performed in order to define the shelf life of the immunomodulator during a 6-month period under refrigeration conditions (5°C ± 3°C). The samples were analyzed by appearance, pH, total protein by UV at 280 nm, SEC, and sterility every three months (0, 3, and 6 months).

## 3. Results and Discussion

The immunomodulators used for the treatment of chronic diseases were analyzed through SEC and MS, in order to demonstrate consistency and peptide distribution stability, unlike standard drugs which contain a single and well-defined compound as the active pharmaceutical ingredient (API) that complies with a quality specification. In biological products such as the immunomodulator peptide studied here, which is obtained from autologous urine, thus, differences in peptide sequences among patients are expected. Therefore, the consistency of the process is determined by the peptide polydispersity given by the molecular mass of distribution of the peptides and the total protein content along with other attributes such as appearance and safety testing.

### 3.1. Size Exclusion Analysis

**Figure 1:**
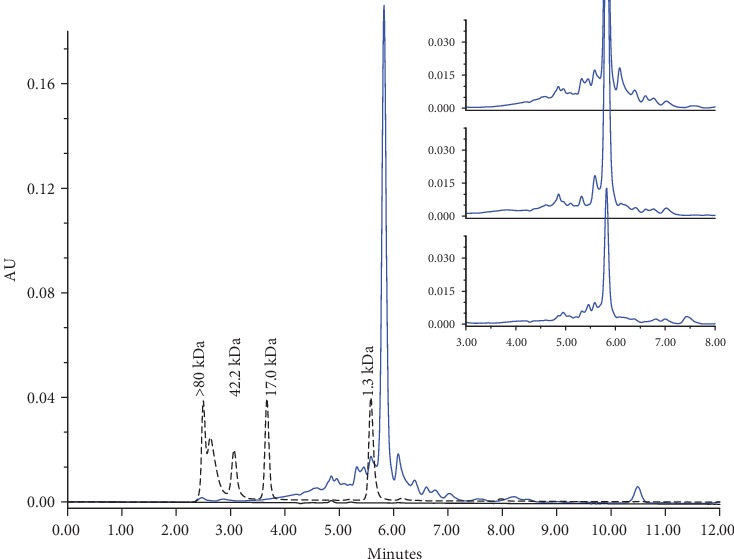
Peptide mass distribution of the immunomodulator by size exclusion chromatography. The main figure shows the characteristic chromatographic profile of the immunomodulator (blue line), the molecular weight marker (dotted line), and matrix (black line). At the right side, it shows the consistency in the mass distribution of three immunomodulators obtained from different volunteers.

Mixture peptide distribution obtained from the immunomodulator showed a peptide size range lower than 17.0 and 1.3 kDa, according to molecular weight markers. A main peak is showed in a retention time of 5.8 min, which corresponds to the formulation excipient (Figure 1). A robust process was designed to extract the immunomodulator from the same source in this case urine of a different volunteer for reinjecting treatment in order to obtain peptides in the expected size range as was showed in the analysis of three independent samples studied by SEC (Figure 1). In these samples, the same range of size was observed but with a different relative abundance of peptide according to the chromatographic profile. The difference depends on the protein concentration, relative abundance, and type of peptides in the urine; it is well known that protein concentration could be increased in donors with some disease and even their urine could present different proteins to those that are normally found [15–17].

### 3.2. Mass Spectrometry Analysis

**Figure 2:**
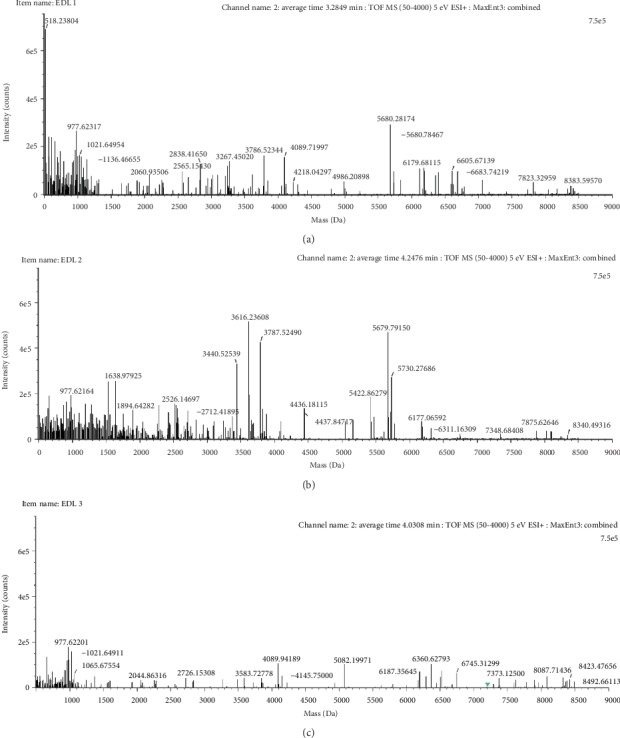
Immunomodulator exact mass distribution spectra obtained from (a) volunteer 1 with allergic rhinitis, (b) volunteer 2 with arthritis rheumatoid, and (c) volunteer 3 with chronic rhinopharyngitis. The horizontal and vertical axes show the mass in daltons and the intensity generated by ionized peptides, respectively.

The mass spectrum criterium was based on the quantitative range of molecular mass of the peptides between 200 and 9000 Da. It confirms, orthogonally, the masses of SEC chromatographic profile. The results showed expected intrinsic differences in peptide abundance among samples. This intrinsic heterogeneity of peptides between volunteers is found in previous studies when screening peptides from urine in order to find diagnostic and prognostic makers, even to use them as therapy in the treatment for some disease [7, 8]. In all cases, the appearance of a high frequency of low molecular weight peptides lower than 2000 Da was found (Figure 2). The mass spectra of the samples from patients showed differences in the abundance of peptides (Figure 2). These mass spectra were obtained from deconvoluted *m*/*z* from reversed-phase chromatograms ([Supplementary-material supplementary-material-1]). This result demonstrated that the origin and abundance of peptides depend on the health status of the donor. Immunomodulators could have sequences of peptides that mimic the epitopes of several antigens that could explain why the immunomodulators could turn on or turn off the innate immune response as universal immunocorrectors. The personalized medicine, such as the present immunomodulator, could contain relevant peptides for the patient from which they were extracted. These peptides could induce a specific immune response that improves the condition of the patient.

### 3.3. Stability Test Analysis

**Figure 3:**
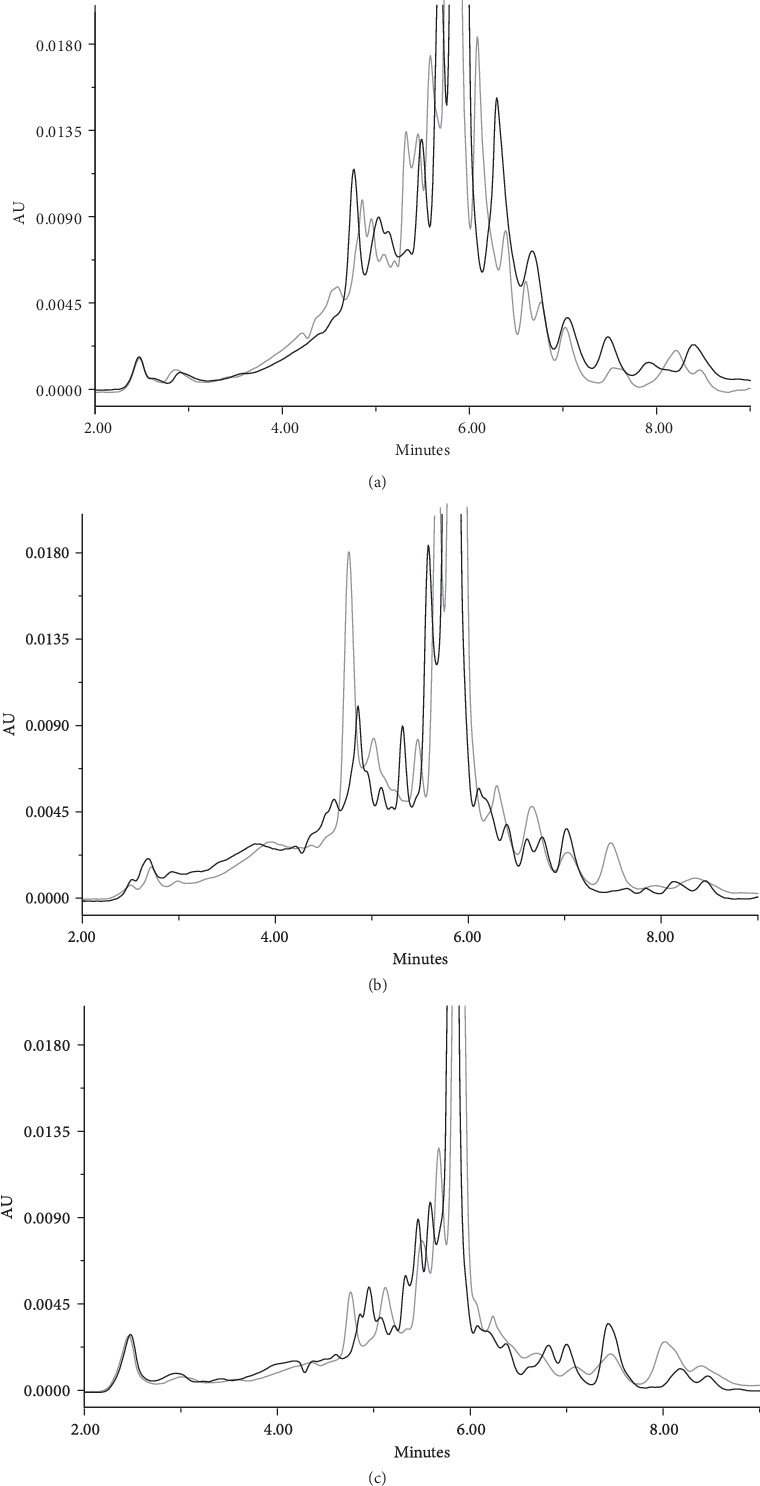
Size distribution profile of immunomodulator product by SEC during stability test at started time (gray line) and after six months (black line). The immunomodulator was produced from (a) volunteer 1, (b) volunteer 2, and (c) volunteer 3.

The shelf life of the immunomodulator was performed during six months, analyzing every three months (Table 1). The results showed consistency in the critical quality attributes evaluated; in all cases, the expected results from each analysis were within the established specifications limits (Table 2). The results from this study allowed to establish shelf life stability of the product. The results by SEC in the independent samples showed the same molecular size distribution and similar chromatographic profile at started time and after six months of storage in refrigeration condition; the total area under the peaks is maintained, which suggests that the peptides are in solution without significant structural changes (Figure 3). Additionally, the size distribution profile remained was confirmed by MS after nine months at refrigeration (Figures [Supplementary-material supplementary-material-1]). The immunomodulator obtained from a volunteer contains a high peptide heterogeneity in terms of size and sequence; even this heterogeneity is greater among volunteers, since it depends on the proteins that have been secreted in the urine. Therefore, the conservation of the peptide distribution is a stability-indicating analysis.

## 4. Conclusion

The consistency in the size distribution profile of complex mixture of peptides obtained by established process is reproducible among different volunteers; it is key to expect the desired response in the treatment of chronic immunological disorders such as allergies of the skin and mucosa, reactive arthritis, and psoriasis. In addition, the product showed to be stable, maintaining its critical quality attributes when it is stored between 5 and 8°C before its use. Therefore, this immunomodulator represents a viable alternative for the treatment of chronic immunological disorders.

## Figures and Tables

**Table 1:** Design of the stability test of immunomodulator.

Test	Method	Time point for sample analysis (months)		
		0	3	6
Appearance	Visual	X	X	X
pH	Potentiometric	X	X	X
Total protein	UV	X	X	X
Mass distribution	SEC	X	X	X
Sterility	Microorganisms culture	X	ND	X

ND: not determined.

**Table 2:** Resume of stability test of the immunomodulator at 5 ± 3°C.

Test	Specification	Results of the analyses in each time								
		0			3			6		
		V1	V2	V3	V1	V2	V3	V1	V2	V3
Appearance	Clear and colorless liquid	C	C	C	C	C	C	C	C	C
pH	5 to 7	6	6	6	7	7	7	7	7	7
Total protein	5.5 to 7.5 mg/mL	6.9	6.5	6.5	6.6	6.5	6.4	7.2	6.8	7.0
Mass distribution	Peptide distribution < 17.0 kDa	C	C	C	C	C	C	C	C	C
Sterility	Sterile	C	C	C	ND	ND	ND	C	C	C

ND: not determined; V: volunteer; C: comply.

## Data Availability

No data were used to support this study.

## Supplementary Materials

Supplementary Materials Figure S1: reversed-phase base peak intensity profile of immunomodulators from volunteer 1 with allergic rhinitis (A), volunteer 2 with rheumatoid arthritis (B), volunteer 3 with chronic rhinopharyngitis (C), and matrix (D). Figure S2: exact mass distribution spectra of immunomodulators after storage at 5 ± 3°C for 9 months.
