# Supplementary material for: Characterization of a Complex Mixture of Immunomodulator Peptides Obtained from Autologous Urine
Source: J Immunol Res. 2020 Apr 2;2020:3683782. doi: 10.1155/2020/3683782 (PMC7154977; doi:10.1155/2020/3683782)
Supplement: Supplementary Materials — Figure S1: reversed-phase base peak intensity profile of immunomodulators from volunteer 1 with allergic rhinitis (A), volunteer 2 with rheumatoid arthritis (B), volunteer 3 with chronic rhinopharyngitis (C), and matrix (D). Figure S2: exact mass distribution spectra of immunomodulators after storage at 5 ± 3°C for 9 months. [file 3683782.f1.pdf]

Supplementary Materials

Characterization of a complex mixture of immunomodulator peptides obtained from autologous urine

Alberto Fragoso[1], Mérida Pedraza-Jiménez[1], Laura Espinoza-González[1], María Luisa Ceja-Mendoza[1], Hugo Sánchez-Mercado[1], Gloria Robles-Pérez[1], Julio Granados[1,2]*, Emilio Medina-Rivero[3]*

[1] Instituto de Alergias y Autoinmunidad Dr. Maximiliano Ruiz Castañeda A.C., Luisa Isabel Campos #16, col. Revolución, Acambay, Edo. de México, C.P. 50300, México.
[2] Departamento de Trasplantes, Instituto Nacional de Ciencias Médicas y Nutrición Salvador Zubirán, Ciudad de México, México.
[3] Unidad de Desarrollo e Investigación en Bioprocesos (UDIBI), Escuela Nacional de Ciencias Biológicas, Instituto Politécnico Nacional, Ciudad de México 11340, México.

Correspondence should be addressed to Emilio Medina-Rivero; emilio.medina@udibi.com.mx and Julio Granados; julgrate@yahoo.com

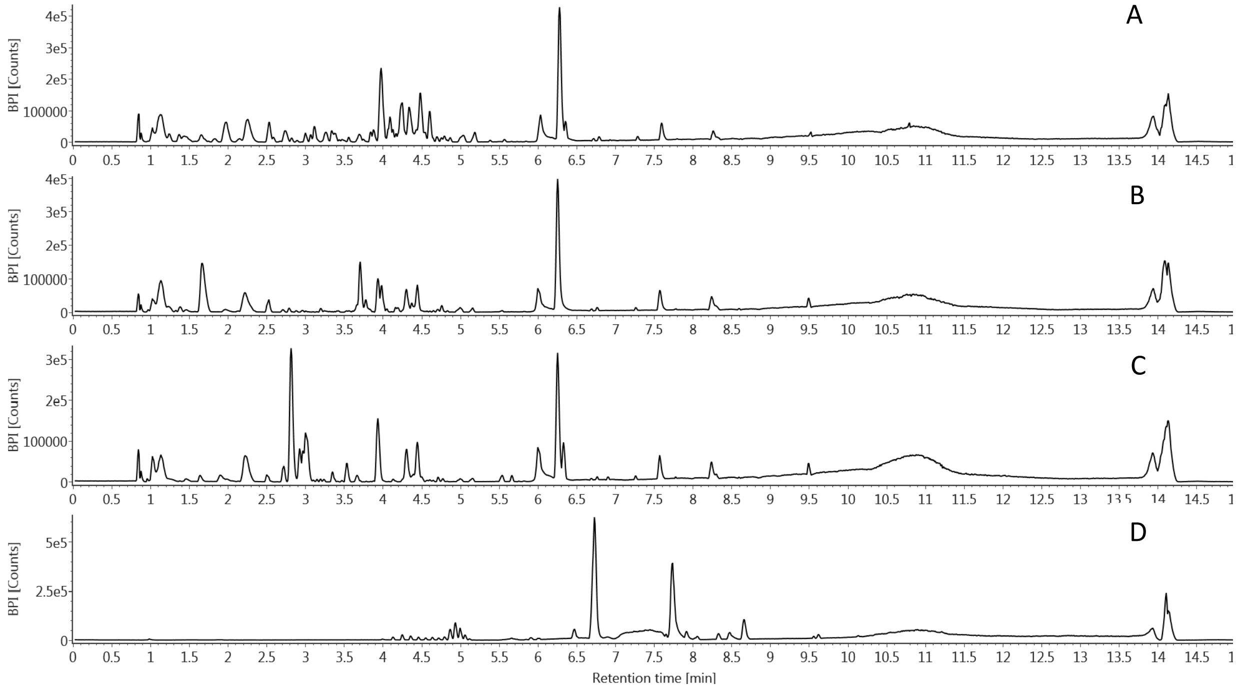

Figure S1. Reversed-phase base peak intensity profile of immunomodulators from volunteer 1 with allergic rhinitis (A), volunteer 2 with rheumatoid arthritis (B), volunteer 3 with chronic rhinopharyngitis (C), and matrix (D).

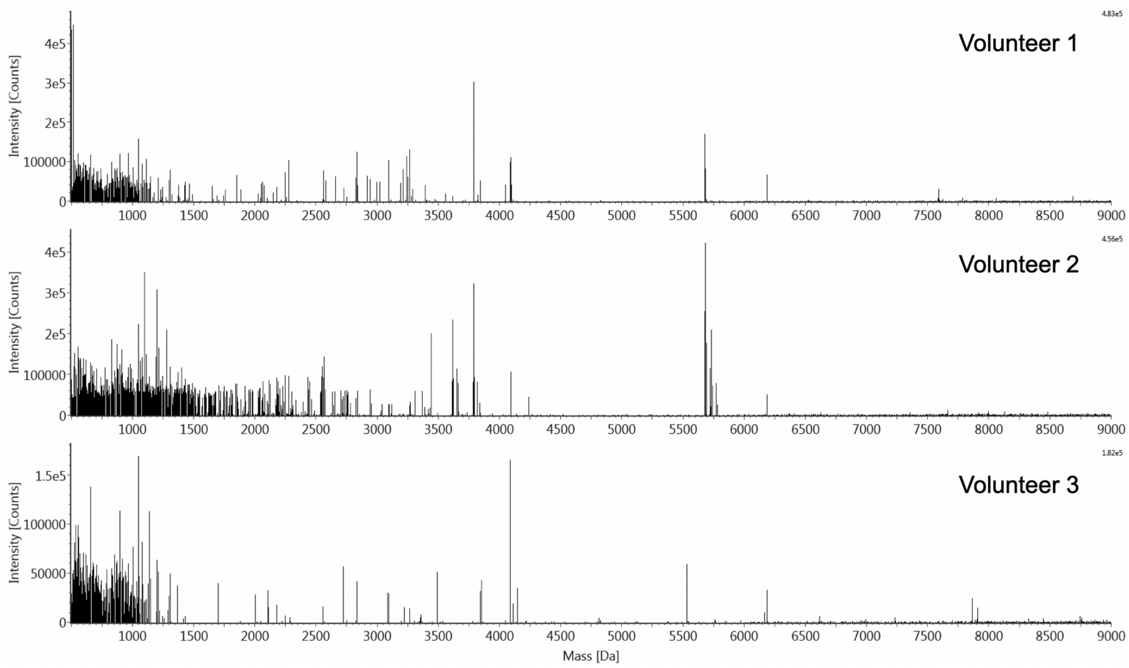

Figure S2. Exact mass distribution spectra of immunomodulators after storage at 5 ± 3 °C for 9 months.
